# Supplementary material for: Branched Chain Amino Acids Are Associated with Physical Performance in Patients with End-Stage Liver Disease
Source: Biomolecules. 2023 May 12;13(5):824. doi: 10.3390/biom13050824 (PMC10216657; doi:10.3390/biom13050824)
Supplement: Supplementary file 1 [file biomolecules-13-00824-s001.zip › biomolecules-2318055-supplementary.pdf]

biomolecules

SUPPLEMENTAL TABLES

Table S1. Multivariable linear regression model of comorbidities predicting plasma BCAA values (μmol/L).

Model variables	β	P value
Hypertension	0.003	0.980
Diabetes	0.222	0.036
Hyperlipidemia	0.055	0.597

β: standardized regression coefficient

Table S2. Multivariable linear regression model of drugs predicting plasma BCAA concentrations (μmol/L) in plasma.

Model variables	β	P value
Statins	0.014	0.902
Diuretics	-0.010	0.938
Vitamin K antagonists	0.023	0.838
Glucose lowering medication	0.262	0.794
Antihypertensives	-0.094	0.925

β: standardized regression coefficient

Table S3. Multivariable linear regression model of age, CPT category, MELD score and plasma BCAA concentrations predicting physical performances.

Model variables	β	P value
**Hand grip strength (kg)**		
Age	0.030	0.791
CPT score	-0.113	0.467
MELD score	-0.017	0.912
Plasma BCAA	-269	0.024
**4 meter walking test (s)**		
Age	-0.129	0.404
CPT score	0.451	0.050
MELD score	-0.133	0.552
Plasma BCAA	-0.033	0.835
**Sit-to-stand test (s)**		
Age	0.031	0.842
CPT score	0.304	0.198
MELD score	0.000	0.998
Plasma BCAA	-0.204	0.259
**Timed up and go test (s)**		
Age	-0.176	0.256
CPT score	0.057	0.814
MELD score	-0.086	0.702
Plasma BCAA	-0.422	0.029
**Standing balance test (points)**		
Age	0.320	0.063
CPT score	0.200	0.470
MELD score	0.082	0.761
Plasma BCAA	0.037	0.840

β: standardized regression coefficient
